# Supplementary material for: Charge Transfer with a Spin. I: A Variational Constrained-CASSCF Framework for Investigating Charge Transfer in the Presence of Spin–Orbit Coupling
Source: J Phys Chem A. 2026 Oct 23;130(44):8923–37. doi: 10.1021/acs.jpca.6c05082 (PMC13750822; doi:10.1021/acs.jpca.6c05082)
Supplement: Supplementary file 1 [file jp6c05082_si_001.pdf]

# Charge Transfer with a Spin. I: A Variational Constrained-CASSCF Framework for Investigating Charge Transfer in the Presence of Spin–Orbit Coupling

Alok Kumar,[†,‡] Zhen Tao,[¶] Joseph E. Subotnik,[‡] and Tian Qiu[*,‡]

†*Department of Chemistry, University of Pennsylvania, Philadelphia, Pennsylvania, 19104, USA*

‡*Department of Chemistry, Princeton University, Princeton, New Jersey, 08540, USA*

¶*Department of Chemistry, University of Rhode Island, Kingston, Rhode Island, 02881, USA*

E-mail: tianq@princeton.edu

# 1 Geometry of phenoxy-phenol (ph-ph) system

C -3.339987 1.236679 -0.292676

C -2.215700 0.395873 -0.461119

C -2.260068 -0.923462 0.044893

C -3.395583 -1.374735 0.696165

C -4.500797 -0.539367 0.860105

C -4.462623 0.765553 0.361575

O -1.170747 0.860673 -1.076240

H -1.404926 -1.560653 -0.084864

H -3.424139 -2.378976 1.078261

H -5.377346 -0.897488 1.367665

H -5.313306 1.410015 0.488105

H -3.290462 2.235719 -0.683604

C 2.967610 -0.964278 -0.250086

C 1.842996 -0.128779 -0.443279

C 1.884680 1.202903 0.030149

C 3.017817 1.671520 0.673563

C 4.123167 0.841308 0.861788

C 4.087496 -0.476001 0.395845

O 0.800605 -0.609058 -1.049392

H 1.029641 1.836307 -0.118287

H 3.044634 2.685260 1.030351

H 4.998118 1.212844 1.362705

H 4.938888 -1.115655 0.540969

H 2.920157 -1.972439 -0.616477

H -0.579299 0.419269 -1.076240
